# Deep Neural Network With a Smooth Monotonic Output Layer for Dynamic Risk Prediction

**DOI:** 10.1002/sim.70401

**Published:** 2026-02-04

**Authors:** Zhiyang Zhou, Yu Deng, Lei Liu, Hongmei Jiang, Yifan Peng, Xiaoyun Yang, Yun Zhao, Hongyan Ning, Norrina B. Allen, John T. Wilkins, Kiang Liu, Donald M. Lloyd‐Jones, Lihui Zhao

**Affiliations:** ^1^ Joseph J. Zilber College of Public Health University of Wisconsin‐Milwaukee Milwaukee WI USA; ^2^ Center for Health Information Partnerships Northwestern University Feinberg School of Medicine Chicago IL USA; ^3^ Center for Biostatistics and Data Science, Institute for Informatics, Data Science, and Biostatistics Washington University in St. Louis St. Louis MO USA; ^4^ Department of Statistics and Data Science Northwestern University Evanston IL USA; ^5^ Department of Population Health Sciences Weill Cornell Medicine New York NY USA; ^6^ Department of Preventive Medicine Northwestern University Feinberg School of Medicine Chicago IL USA; ^7^ Department of Computer Science University of California, Santa Barbara Santa Barbara CA USA; ^8^ Chobanian & Avedisian School of Medicine Boston University Boston MA USA

**Keywords:** B‐spline, cardiovascular disease, dynamic risk prediction, longitudinal data analysis, survival analysis

## Abstract

Risk prediction is a key component of survival analysis across various fields, including medicine, public health, economics, engineering, and others. The fundamental concern of risk prediction lies in the joint distribution of risk factors and the time to event. The recent success of survival analysis has already been extended to dynamic risk prediction, which incorporates multiple longitudinal observations into predictive models. However, existing methods often rely on parametric model assumptions or discretely approximate survival functions, potentially introducing more bias in predictions. To address these limitations, we introduce a deep neural network featuring a novel output layer termed the Smooth Monotonic Output Layer (SMOL). This model avoids discretization as well as parametric model assumptions. At its core, SMOL takes a general vector as the input and constructs a monotonic, differentiable function via B‐splines. Employing SMOL as the output layer allows for direct, nonparametric estimation of monotonic functions of interest, such as survival and cumulative distribution functions. We performed extensive experiments utilizing data from the Cardiovascular Disease Lifetime Risk Pooling Project (LRPP), which harmonized individual data from multiple longitudinal community‐based cardiovascular disease (CVD) studies. Our results demonstrate that the proposed approach achieves state‐of‐the‐art accuracy in predicting individual‐level risk for atherosclerotic CVD.

## 1 Introduction

Cardiovascular disease (CVD), encompassing a range of heart and blood vessel disorders, is the leading cause of death worldwide, accounting for around 18 million deaths each year [1]. Although CVD is preventable through lifestyle changes and medical interventions, a large number of individuals with high CVD risk remain undetected and hence untreated, resulting in poor health outcomes. Thus, there is an urgent need to assess individualized risk for CVD. The Cox proportional hazards model (CPH) [2], the accelerated failure time model [3] and their numerous variants have widely served for such purposes. Traditionally, even when risk factors are repeatedly measured over time, most predictive models merely utilize the information at a single time point (usually the initial/last visit). As a result, they fail to exploit the full breadth of temporal information, limiting their predictive accuracy. This limitation is severe for predicting CVD risk. It has been shown that 90% of CVD events are attributable to longitudinal, modifiable risk factors [4, 5]. Also, the evolution and cumulative burden of CVD risk factors are associated with the onset of CVD events [6, 7, 8]. Therefore, it is critical to incorporate longitudinal information in order to predict real‐time CVD risks, which gives rise to dynamic risk prediction.

Our research is motivated by data from the CVD Lifetime Risk Pooling Project (LRPP) [9], a large‐scale initiative that harmonizes individual‐level data from twenty community‐based observational CVD studies. This dataset comprises more than 200 000 participants and includes adjudicated CVD events with long‐term follow‐up on vital status. Crucially, it provides rich longitudinal data on repeatedly measured CVD risk factors, offering a rich resource for developing dynamic risk prediction models tailored to CVD.

Methods for dynamic risk prediction usually fall into three main streams: The landmarking (LM), joint modeling (JM), and deep learning. Among them, LM is less complicated in implementation and interpretation [10]. It fits a survival model for subjects who are still at risk at the predetermined landmark time point. Rather than exploring the entire longitudinal history, it is conditional on the available information up to the landmark time. Consequently, the prediction from LM is not “fully dynamic”: Only a subset of longitudinal observations is utilized. Predictions are thus issued merely at the prespecified landmark time. The framework of JM consists of multiple submodels: Each of them corresponds to one longitudinal/survival process, sharing functions of fixed and random effects. Learning the joint distribution of longitudinal and survival processes, JM allows predictions to be made at any time points of interest [11]. Optimizing the complex likelihood, JM faces severe computational challenges, especially when there are multiple longitudinal risk factors [12]. Also, JM is subject to parametric model assumptions. While recent developments allow for nonparametric modeling of the longitudinal submodel, the survival submodel commonly retains the proportional hazards assumption [13, 14, 15, 16]. Comprehensive reviews of JM methods are available in the literature [17, 18, 19, 20]. In addition, empirical comparisons between JM and LM have been conducted to evaluate their respective strengths and limitations [21].

By contrast, involving fewer delicate assumptions, deep learning methods are less likely to suffer from the model misspecification. In recent decades, they have gained increasing acceptance and shown their competitiveness in risk prediction; see, for example, DeepSurv [22], Cox‐nnet [23], DeepHit [24], Nnet‐survival [25], Cox‐MLP [26], Cox‐Time [26], Soden [27], and SuMo‐net [28]. However, most of them are not designed for longitudinal measurements and therefore cannot accommodate the dynamic risk prediction. Several exceptions have emerged, including Dynamic‐DeepHit (DDH) [29], Match‐Net [30], Recurrent Deep Survival Machine (RDSM) [31], and DeepJoint [32]. Among these, RDSM and DeepJoint require more careful specification of model components: RDSM leverages longitudinal measurements to estimate parameters of a mixture model with log‐normal or Weibull components, while DeepJoint extends DeepSurv by jointly modeling CPH along with longitudinal and missingness processes. In contrast, DDH and Match‐Net provide nonparametric estimators of survival probabilities. Specifically, DDH integrates a recurrent neural network (RNN, such as long short‐term memory (LSTM) [33] and gated recurrent units (GRU) [34]) with a temporal attention mechanism (TAM) [35] and a feed‐forward neural network (FNN) [36]. Match‐Net, on the other hand, employs a convolutional neural network [37] to extract representations from longitudinal observations. With the softmax [38] output layer, both DDH and Match‐Net discretize the time domain and approximate the survival function by a probability mass function, requiring that the event of interest must occur before a preset time point.

Our contribution is a novel type of output layer for neural networks, referred to as the Smooth Monotonic Output Layer (SMOL). SMOL is general‐purpose: It is attachable to arbitrary neural networks if one would like to estimate a monotonic, differentiable function.

A growing body of literature has focused on enforcing monotonicity in neural networks for risk prediction. Common strategies include the use of nested hyperbolic tangent functions [28, 39, 40, 41] or indefinite integrals of positive‐valued functions [42]. In contrast to these efforts, SMOL is based on basis splines (B‐splines) [43], which provides a flexible and widely accepted framework for approximating functions. In this work, for the prediction of CVD risk, we adopt DDH as the backbone due to its competitive discriminative performance and accessible source code. We substitute SMOL for DDH's original output layer, resulting in a new model, DDH‐SMOL. DDH‐SMOL produces a monotonic, differentiable approximation of the survival function, without imposing parametric model assumptions or requiring the event of interest to occur within a fixed time horizon.

The remaining portion of this paper is organized as follows. Section 2 formalizes the dynamic risk prediction by elaborating the target survival function. The architecture of DDH‐SMOL is given in Section 3, where SMOL is also explicitly defined. In Section 4, we apply the proposal to CVD risk prediction and compare the results with those from benchmarks. Section 5 presents a simulation study to evaluate the robustness and efficiency of DDH‐SMOL compared to existing approaches. Section 6 concludes the paper and discusses potential modifications. More theoretical details are relegated to the Appendix.

## 2 Problem Formulation

### 2.1 Time‐to‐Event Data With Longitudinal Observations

**FIGURE 1:**
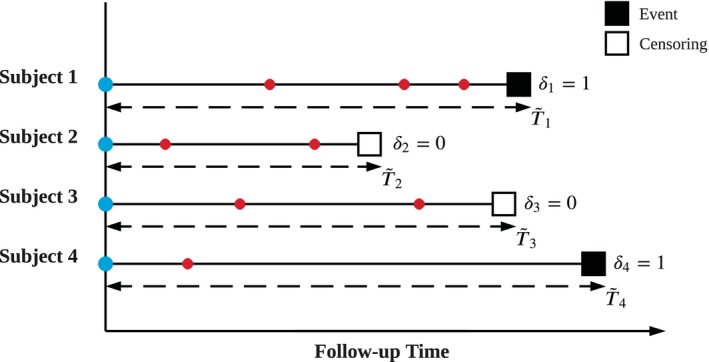
A demo of right‐censored time‐to‐event data with longitudinal measurements. The event and censoring are labeled with solid and hollow boxes, respectively. The follow‐up time is the elapsed time since the start point of data collection. Colored dots indicate visits: blue ones correspond to the initial visits, all aligned such that they occur at (follow‐up) time 0; red ones correspond to subsequent visits. At each visit, $d_{x}$ time‐varying risk factors are measured. Additionally, at the initial visit, $d_{z}$ time‐invariant risk factors are measured too. Missingness may occur for some subjects at certain visits for specific risk factors.

Henceforth, we adopt the follow‐up time scale by aligning time points such that the initial baseline visit of each subject happens at time 0. Denote by $T_{\max}>0$ the maximum (follow‐up) time of interest. We are interested in observations in $\left[0,T_{\max}\right]$, though we do NOT impose the assumption that all subjects should encounter the event within $\left[0,T_{\max}\right]$. For subject i, i=1,…,n, time‐invariant risk factors are recorded as $z_{i}={\left[z_{i,1},\ldots ,z_{i,d_{z}}\right]}^{⊤}\in {\mathbb{R}}^{d_{z}}$, while $d_{x}$‐vector $x_{i,j}={\left[x_{i,j,1},\ldots ,x_{i,j,d_{x}}\right]}^{⊤}$ contains the values of time‐varying risk factors measured at the jth visit. Notably, risk factors may not be measured altogether at each visit; there may be some missing entries in $z_{i}$ or $x_{i,j}$ for certain values of i and j. Following DDH, we apply a simple imputation by replacing missing values with placeholders such as zeros or means and additionally introduce the binary missingness mask, that is, indicator vectors that specify which entries in $z_{i}$ or $x_{i,j}$ are unobserved. Specifically, we define indicator vectors $m_{i,j}={\left[m_{i,1}^{z},\ldots ,m_{i,d_{z}}^{z},m_{i,j,1}^{x},\ldots ,m_{i,j,d_{x}}^{x}\right]}^{⊤}\in {\mathbb{R}}^{d_{z}+d_{x}}$, where $m_{i,k_{z}}^{z}=1(z_{i,k_{z}}\text{is not observed})$, $k_{z}=1,\ldots ,d_{z}$, and $m_{i,j,k_{x}}^{x}=1(x_{i,j,k_{x}}\text{is not observed})$, $k_{x}=1,\ldots ,d_{x}$, with 1(·) denoting the indicator function. Let $\tau_{i,j}$ be the jth visit time of subject i. Noting that these observational times are not necessarily regularly spaced (see the colored dots in Figure 1), we include the gap between each pair of adjacent visit times, that is, $\Delta \tau_{i,1}=0$ and $\Delta \tau_{i,j}=\tau_{i,j}-\tau_{i,j-1}$ for j≥2. All the information for subject i until time t are summarized into a set

(1) $$y_{i,t}=\{z_{i},x_{i,j},m_{i,j},\Delta \tau_{i,j}:j=1,\ldots ,J_{i}(t)\},$$

where $J_{i}(t)$ represents the number of visits of subject i on and before time t.

For the ith subject, denote the event and censoring times by $T_{i}$ and $C_{i}$, respectively. We make the usual assumption that the censoring is noninformative. For subject i, the observable survival data is $\left({\tilde{T}}_{i},\delta_{i}\right)$, where time ${\tilde{T}}_{i}=\min(T_{i},C_{i})$, the smaller of $T_{i}$ and $C_{i}$, and the event indicator $\delta_{i}=1(T_{i}\leq C_{i})$ takes value one if the event is observed and zero for censoring.

### 2.2 Survival Function and Cumulative Incidence Function

Given $0\leq t<s\leq T_{\max}$, for subject i, dynamic risk prediction is focused on the (conditional) survival function, that is, the probability that subject i surviving by time t will survive past time s, conditional on $y_{i,t}$ in (1). Specifically, the survival function is defined as

(2) $$S(s|y_{i,t},t)=\mathrm{Pr}(T_{i}>s|y_{i,t},T_{i}>t),$$

which is descending with respect to s. In addition, $S(T_{\max}|y_{i,t},t)$ converges to zero as $T_{\max}$ goes to infinity. Both DDH and Match‐Net forced $S(T_{\max}|y_{i,t},t)$ to be zero for all $t\in [0,T_{\max})$, whereas we do not impose such a restriction. Corresponding to the survival function (2), the cumulative incidence function is

(3) $$F(s|y_{i,t},t)=1-S(s|y_{i,t},t)$$

which is the probability for the ith subject to experience the event on or before time s, conditioning on historical observations up to time t.

## 3 Model Description

**FIGURE 2:**
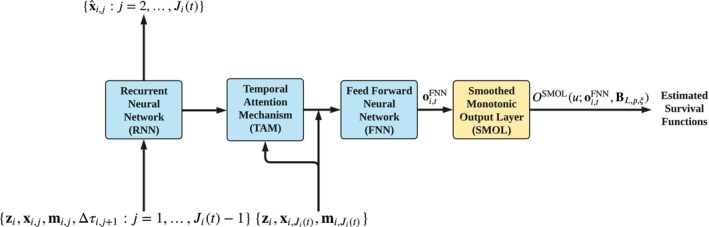
Architecture of DDH‐SMOL, that is, the concatenation of DDH (with the softmax output layer removed) and SMOL. Passing $o_{i,t}^{\text{FNN}}$, the output of RNN module, to SMOL, one may yield $O_{\text{SMOL}}(u;o_{i,t}^{\text{FNN}},B_{L,p,\xi })$ in (10), a monotonic differentiable function of u.

In this section we construct a deep neural network by substituting SMOL for the original output layer of DDH (Figure 2). The resulting DDH‐SMOL is trained by minimizing the penalized negative log‐likelihood.

### 3.1 DDH‐SMOL

DDH‐SMOL is composed of two core components: the backbone architecture adapted from DDH and SMOL, which replaces the original output layer in DDH. Specifically, the backbone architecture includes a sequence of three subnetworks (see the first three blue bricks in Figure 2):

1. A RNN (such as LSTM and GRU) for handling sparse and potentially incomplete longitudinal measurements. It generates both a hidden representation of the sequence, say $h_{i,j}$, and a prediction for $x_{i,j}$, say ${\hat{x}}_{i,j}={\left[{\hat{x}}_{i,j,1},\ldots ,{\hat{x}}_{i,j,d_{x}}\right]}^{⊤}$. Feeding the combined input $y_{i,t}$ (as defined in (1)) into, for example, GRU, vectors $h_{i,j}$ and ${\hat{x}}_{i,j}$ are derived as follows:
(4) $$\begin{array}{ll} a_{i,j} & =\text{sigmoid}(W_{ah}h_{i,j-1}+W_{ay}{\left[z_{i}^{⊤},x_{i,j}^{⊤},m_{i,j}^{⊤}\right]}^{⊤}+b_{a}),\\ c_{i,j} & =\text{sigmoid}(W_{ch}h_{i,j-1}+W_{cy}{\left[z_{i}^{⊤},x_{i,j}^{⊤},m_{i,j}^{⊤}\right]}^{⊤}+b_{c}),\\ {\tilde{h}}_{i,j} & =\tanh(W_{hh}(c_{i,j}⊙h_{i,j-1})+W_{hy}{\left[z_{i}^{⊤},x_{i,j}^{⊤},m_{i,j}^{⊤}\right]}^{⊤}+b_{h}),\\ h_{i,j} & =(1-a_{i,j})⊙h_{i,j-1}+a_{i,j}⊙{\tilde{h}}_{i,j}, \end{array}$$
(5) $$\begin{array}{ll} {\hat{x}}_{i,j} & =W_{xh}h_{i,j}+b_{x}, \end{array}$$
  where ⊙ represents the Hadamard product, W's are weight matrices, and b's are bias vectors. The sigmoid and hyperbolic tangent functions, that is, sigmoid(·) and tanh(·), are both applied entry‐wise. The hidden state is initialized as $h_{i,0}=0$ for all i. While GRU is illustrated here, other RNN variants such as LSTM can also be used in this module.
2. A TAM for determining which parts of previous longitudinal measurements are most relevant for the current prediction. This module outputs a weighted sum of past hidden states $h_{i,j}$ given by (4), that is,
(6) $$o_{i,t}^{\text{TAM}}=\overset{J_{i}(t)-1}{\underset{j=1}{\sum }}\frac{\exp\{f_{\text{TAM}}(h_{i,j},z_{i},x_{i,J_{i}(t)},m_{i,J_{i}(t)})\}}{\sum_{\ell =1}^{J_{i}(t)-1}\exp\{f_{\text{TAM}}(h_{i,\ell },z_{i},x_{i,J_{i}(t)},m_{i,J_{i}(t)})\}}h_{i,j},$$
  where $f_{\text{TAM}}(\cdot )$ represents a two‐layer FNN that outputs a scalar $f_{\text{TAM}}(h_{i,j},z_{i},x_{i,J_{i}(t)},m_{i,J_{i}(t)})$ scoring the importance of each previous visit up to time t.
3. An FNN composed of fully‐connected layers for capturing latent patterns from the weighted representation of longitudinal history. Taking both $o_{i,t}^{\text{TAM}}$ (6) and the last measurement $\left\{z_{i},x_{i,J_{i}(t)},m_{i,J_{i}(t)}\right\}$ as the input, this module outputs a real‐valued (L+p)‐vector relying on t, say $o_{i,t}^{\text{FNN}}$, where integers L≥1 and p≥2 are both tunable hyperparameters. $o_{i,t}^{\text{FNN}}$ serves as a compact and informative summary of all available information on subject i up to time t.

We then elaborate the mechanism of SMOL. It first defines p‐degree (univariate) B‐splines $B_{\ell ,p,\xi }(\cdot )$, ℓ=1,…,L+p, with an ascending knot sequence

(7) $$\xi =\{\xi_{1},\ldots ,\xi_{L+2p+1}\},$$

where $\xi_{1}\leq \cdots \leq \xi_{p}<\xi_{p+1}=0<\cdots <\xi_{L+p+1}=T_{\max}<\xi_{L+p+2}\leq \cdots \leq \xi_{L+2p+1}$. Please refer to the appendix for the detailed expression of $B_{\ell ,p,\xi }(\cdot )$. Denoting by $o_{i,t,\ell }^{\text{FNN}}$ the ℓth entry of $o_{i,t}^{\text{FNN}}$, apply a function ϕ:ℝ→ℝ to $o_{i,t}^{\text{FNN}}$ entry‐wise such that

(8) $$\phi (o_{i,t,\ell }^{\text{FNN}})>0\;\text{for}\;\ell =2,\ldots ,L+p.$$

Notably, such ϕ is far from unique. To mitigate the risk of exploding gradients, we consider an alternative to the exponential function by applying the shifted rectified linear unit [44] to all but the first entry of $o_{i,t}^{\text{FNN}}$. In particular, we define ϕ as follows:

(9) $$\phi (o_{i,t,\ell }^{\text{FNN}})=\left\{\begin{array}{ll} o_{i,t,\ell }^{\text{FNN}}\; & \ell =1\\ \max\{0,\overset{\text{FNN}}{\underset{i,t,\ell }{o}}\}+\varepsilon_{\phi }\; & \ell =2,\ldots ,L+p, \end{array}\right.$$

where $\varepsilon_{\phi }$ is a pre‐determined tiny positive number; see Section 4.3 for the value of $\varepsilon_{\phi }$ in our numerical experiments. Provided that the condition (8) is satisfied, the following function is guaranteed to be strictly increasing with respect to u:

(10) $$O_{\text{SMOL}}(u;o_{i,t}^{\text{FNN}},B_{L,p,\xi })=\overset{L+p}{\underset{\ell =1}{\sum }}\left\{\overset{\ell }{\underset{k=1}{\sum }}\phi (o_{i,t,k}^{\text{FNN}})\right\}B_{\ell ,p,\xi }(u)$$

in which $B_{L,p,\xi }$ is the set of B‐splines $B_{\ell ,p,\xi }(\cdot )$, ℓ=1,…,L+p. (10) serves as the foundation for our subsequently constructed estimator of the survival function (2). Its monotonicity is formally stated in Proposition 1, with the proof relegated to the appendix. In addition, with respect to u, the (p−1)th order partial derivative of (10) is continuous [45].

Proposition 1 *If* p≥2, *then* $O_{\text{SMOL}}(u;o_{i,t}^{\text{FNN}},B_{L,p,\xi })$ *in* (*10*) *is strictly increasing with respect to* u *in* $\left[0,T_{\max}\right]$.

### 3.2 Estimator of Survival Function

Fixing t, we note from (10) and Proposition 1 that $O_{\text{SMOL}}(t;o_{i,t}^{\text{FNN}},B_{L,p,\xi })-O_{\text{SMOL}}(s;o_{i,t}^{\text{FNN}},B_{L,p,\xi })$ is negative and strictly decreasing with respect to s for all s>t. This property motivates the following estimator for the survival function (2):

(11) $$\begin{array}{cc} \hat{S}(s|y_{i,t},t) & =\exp\left\{O_{\text{SMOL}}(t;o_{i,t}^{\text{FNN}},B_{L,p,\xi })\right.\left.-O_{\text{SMOL}}(s;o_{i,t}^{\text{FNN}},B_{L,p,\xi })\right\}\\ & =\exp\left[\overset{L+p}{\underset{k=1}{\sum }}\phi (o_{i,t,k}^{\text{FNN}})\overset{L+p}{\underset{\ell =k}{\sum }}\{B_{\ell ,p,\xi }(t)-B_{\ell ,p,\xi }(s)\}\right] \end{array}$$

for given $0\leq t<s\leq T_{\max}$ and historical observations $y_{i,t}$. The corresponding estimator of the cumulative incidence function (3) is then given by

(12) $$\hat{F}(s|y_{i,t},t)=1-\hat{S}(s|y_{i,t},t).$$

Notably, for all $0\leq t<s\leq T_{\max}$, the estimator (11) lies strictly in the interval (0,1) and is strictly decreasing in s. Furthermore, when ϕ is specified as in (9), $\hat{S}(T_{\max}|y_{i,t},t)$ converges to zero as both $T_{\max}$ and L tend to infinity. This demonstrates that the proposed estimator (11) preserves key properties of survival function (2).

### 3.3 Loss Function

The total loss is defined as

(13) $$\tilde{ℒ}=ℒ+\lambda_{1}{𝒫}_{1}+\lambda_{2}{𝒫}_{2}+\lambda_{3}{𝒫}_{3},$$

where the first term on the right‐hand side, ℒ, is the negative (observed‐data) log‐likelihood, while the remaining three are penalties enhancing the overall performance; $\lambda_{1}$, $\lambda_{2}$, and $\lambda_{3}$ are all positive hyper‐parameters to be tuned. Specifically, thanks to the differentiability (with respect to s) of $\hat{F}(s;y_{i,t},t)$ in (12),

$$\begin{array}{cc} ℒ & =-\frac{1}{n}\overset{n}{\underset{i=1}{\sum }}\left\{\delta_{i}\ln\frac{\partial \hat{F}(s|y_{i,\tau_{i,J_{i}({\tilde{T}}_{i})}},\tau_{i,J_{i}({\tilde{T}}_{i})})}{\partial s}{|}_{s={\tilde{T}}_{i}}\right.\\ & \;\left.+(1-\delta_{i})\ln\hat{S}({\tilde{T}}_{i}|y_{i,\tau_{i,J_{i}({\tilde{T}}_{i})}},\tau_{i,J_{i}({\tilde{T}}_{i})})\right\}\\ & =-\frac{1}{n}\overset{n}{\underset{i=1}{\sum }}\left[\overset{L+p}{\underset{k=1}{\sum }}\phi (o_{i,{\tilde{T}}_{i},k}^{\text{FNN}})\overset{L+p}{\underset{\ell =k}{\sum }}\{B_{\ell ,p,\xi }(\tau_{i,J_{i}({\tilde{T}}_{i})})-B_{\ell ,p,\xi }({\tilde{T}}_{i})\}\right.\\ & \;\left.+\delta_{i}\ln\left\{\overset{L+p}{\underset{k=1}{\sum }}\phi (o_{i,{\tilde{T}}_{i},k}^{\text{FNN}})\overset{L+p}{\underset{\ell =k}{\sum }}\frac{\partial B_{\ell ,p,\xi }(u)}{\partial u}{|}_{u={\tilde{T}}_{i}}\right\}\right]. \end{array}$$

The three penalty terms focus on distinct aspects. Inherited from DDH, ${𝒫}_{1}$ compares the risks of paired subjects at times elapsed since their last measurements: it is believed that a subject who survives longer since his/her last measurement has a higher survival probability (or a lower risk). A violation of this concordance may lead to a large value of ${𝒫}_{1}$. Going through all $\left(i_{1},i_{2}\right)$‐pairs with ${\tilde{T}}_{i_{1}}-\tau_{i_{1},J_{i_{1}}({\tilde{T}}_{i_{1}})}<{\tilde{T}}_{i_{2}}-\tau_{i_{2},J_{i_{2}}({\tilde{T}}_{i_{2}})}$, ${𝒫}_{1}$ is defined as

$$\begin{array}{ll} {𝒫}_{1}=\frac{1}{n^{2}}\underset{1\leq i_{1},i_{2}\leq n}{\sum } & 1(\delta_{i_{1}}=1,\;{\tilde{T}}_{i_{1}}-\tau_{i_{1},J_{i_{1}}({\tilde{T}}_{i_{1}})}<{\tilde{T}}_{i_{2}}-\tau_{i_{2},J_{i_{2}}({\tilde{T}}_{i_{2}})})\\ & \exp\{\hat{S}({\tilde{T}}_{i_{1}}|y_{i_{1},\tau_{i_{1},J_{i_{1}}({\tilde{T}}_{i_{1}})}},\tau_{i_{1},J_{i_{1}}({\tilde{T}}_{i_{1}})})-\hat{S}({\tilde{T}}_{i_{1}}-\tau_{i_{1},J_{i_{1}}({\tilde{T}}_{i_{1}})}\\ & \;+\tau_{i_{2},J_{i_{2}}({\tilde{T}}_{i_{2}})}|y_{i_{2},\tau_{i_{2},J_{i_{2}}({\tilde{T}}_{i_{2}})}},\tau_{i_{2},J_{i_{2}}({\tilde{T}}_{i_{2}})})\}. \end{array}$$

Recalling ${\hat{x}}_{i,j}$ in (5),

(14) $${𝒫}_{2}=\frac{1}{n}\overset{n}{\underset{i=1}{\sum }}\overset{J_{i}({\tilde{T}}_{i})}{\underset{j=2}{\sum }}\overset{d_{x}}{\underset{\ell =1}{\sum }}(1-m_{i,j,\ell }^{x})\zeta (x_{i,j,\ell },{\hat{x}}_{i,j,\ell })$$

reflects the error in predicting $x_{i,j}=[x_{i,j,1},\ldots ,x_{i,j,d_{x}}]$, where $\zeta (x_{i,j,\ell },{\hat{x}}_{i,j,\ell })$ is defined as ${\left(x_{i,j,\ell }-{\hat{x}}_{i,j,\ell }\right)}^{2}$ for continuous time‐varying risk factors and as $-x_{i,j,\ell }\ln({\hat{x}}_{i,j,\ell }+\varepsilon_{\zeta })-(1-x_{i,j,\ell })\ln(1-{\hat{x}}_{i,j,\ell }+\varepsilon_{\zeta })$ for binary risk factors taking values from {0,1}, with a small positive $\varepsilon_{\zeta }$ predetermined to ensure the feasibility of computation. The remaining

$${𝒫}_{3}=\frac{1}{n}\overset{n}{\underset{i=1}{\sum }}\int_{0}^{T_{\max}}{\left\{\overset{L+p}{\underset{k=1}{\sum }}\phi (o_{i,{\tilde{T}}_{i},k}^{\text{FNN}})\overset{L+p}{\underset{\ell =k}{\sum }}\frac{\partial^{2}B_{\ell ,p,\xi }(u)}{\partial u^{2}}\right\}}^{2}du$$

is the squared $L_{2}$‐norm of the second order derivative of functional output of SMOL. Such a term is commonly adopted to penalize the roughness of functions [46].

## 4 Application

### 4.1 Dataset

Following the 2013 American College of Cardiology/American Heart Association Guideline on the Assessment of Cardiovascular Risk (abbr. the 2013 ACC/AHA CVR Guideline) [47], we used individual data from five community‐based studies, including the Cardiovascular Health Study, the Atherosclerosis Risk in Communities Study, the Coronary Artery Risk Development in Young Adults Study, the Framingham Original Study, and the Framingham Offspring Study, which are all parts of the LRPP. For each subject, the event of interest was the first atherosclerotic cardiovascular disease (ASCVD) event, defined as nonfatal myocardial infarction or coronary heart disease (CHD) death or fatal or nonfatal stroke. Further screening was carried out: records were kept if they were (1) measured at age 40 to 79 and (2) free of ASCVD‐relevant preconditions. Risk factors, screened out by the 2013 ACC/AHA CVR Guideline, were the enrollment age (time‐invariant), systolic blood pressure (time‐varying), total cholesterol (time‐varying), high‐density lipoprotein cholesterol (time‐varying), anti‐hypertensive medication (time‐varying), current smoking status (time‐varying), and diabetes medication (time‐varying). Following the 2013 ACC/AHA CVR Guideline, we split the screened pooled data by sex (female/male) and race (white/black) and analyzed the resulting four sub‐datasets through multiple approaches; see Section 4.2. Descriptive statistics of these four sub‐datasets were summarized in Table 1.

**TABLE 1:** Descriptive statistics of the four ASCVD datasets described in Section 4.1, involving risk factors Age0—enrollment age (time‐invariant, continuous), HDLCHL—high‐density lipoprotein cholesterol (time‐varying, continuous), TOTCHL—total cholesterol (time‐varying, continuous), SBP—systolic blood pressure (time‐varying, continuous), RXHRT—anti‐hypertensive medication (time‐varying, binary), HXDIAB—diabetes medication (time‐varying, binary), and SMOKER—current smoking status (time‐varying, binary).

Dataset	Number of		Follow‐up year	Risk factor	Value
	subjects	events	mean (sd)		mean (sd)
White female	13847	2929	22.0 (10.5)	Age0, year	52.5 (10.3)
				HDLCHL, mg/dL	57.1 (17.6)
				TOTCHL, mg/dL	224.5 (45.1)
				SBP, mmHg	129.9 (22.0)
				RXHYP, %	27.0 (44.4)
				HXDIAB, %	3.2 (17.7)
				SMOKER, %	21.6 (41.2)
White male	11516	3409	19.5 (9.9)	Age0, year	52.1 (9.9)
				HDLCHL, mg/dL	43.7 (13.4)
				TOTCHL, mg/dL	211.7 (41.6)
				SBP, mmHg	130.1 (19.4)
				RXHYP, %	23.6 (42.5)
				HXDIAB, %	4.1 (19.9)
				SMOKER, %	27.2 (44.5)
Black female	3628	617	16.0 (6.9)	Age0, year	52.0 (9.5)
				HDLCHL, mg/dL	57.6 (18.2)
				TOTCHL, mg/dL	204.7 (42.1)
				SBP, mmHg	128.7 (20.9)
				RXHYP, %	48.2 (50.0)
				HXDIAB, %	13.5 (34.2)
				SMOKER, %	18.6 (38.9)
Black male	2426	513	14.4 (7.1)	Age0, year	51.8 (9.3)
				HDLCHL, mg/dL	50.0 (17.2)
				TOTCHL, mg/dL	196.9 (40.9)
				SBP, mmHg	128.8 (20.0)
				RXHYP, %	37.8 (48.5)
				HXDIAB, %	12.7 (33.3)
				SMOKER, %	30.0 (45.8)

### 4.2 Benchmarks

We included DDH as one benchmark. The reason was bifold: (1) an empirical comparison between DDH and DDH‐SMOL directly reflected the utility of SMOL; (2) DDH illustrated its overwhelming advantage over a series of state‐of‐the‐art methods [29], including the random survival forests [48] and JM implemented through Markov chain Monte Carlo samplers [49] which was our second benchmark. The 2013 ACC/AHA CVR Guideline developed the sex‐ and race‐specific Pooled Cohort Equations based on static CPH models. Accordingly, we included static CPH models as a benchmark in our analysis. Specifically, we used only the initial measurements of risk factors to fit CPH models and compute survival probabilities via R package survival [50]. To approximate the desired conditional survival probability in (2), we then took the ratio of relevant survival probabilities [51]. In this way, CPH actually yielded a non‐dynamic prediction, as it relied solely on baseline information rather than time‐varying measurements.

### 4.3 Hyperparameters

We adopted (9) as the activation function ϕ used in functions (10), (11), and (12). Tiny positive numbers, such as $\varepsilon_{\phi }$ in (9) and $\varepsilon_{\zeta }$ in (14), were set to $10^{-8}$, consistent with the source code of DDH at GitHub (https://github.com/chl8856/Dynamic‐DeepHit).

To ensure a fair comparison between DDH and DDH‐SMOL, they were both trained by backpropagation via the Adam optimizer [52] and shared the identical candidate values for common hyperparameters, including the dropout rate, learning rate, mini‐batch size, activation functions, number of hidden layers, and number of nodes of each subnetwork. For specific values, please refer to Appendix B of the paper on DDH [29].

Hyper‐parameters unique to DDH‐SMOL, namely L and p, took values from {10,30,50,100,300,500} and {3,5,7,9}, respectively. Candidate pools for $\log_{10}\lambda_{1}$, $\log_{10}\lambda_{2}$, and $\log_{10}\lambda_{3}$ (with $\lambda_{1}$, $\lambda_{2}$, and $\lambda_{3}$ all defined in (13)) were three continuums [−1,1], [−3,1], and [0,4], respectively. For simplicity, the knots of B‐spline in (7) were evenly spaced, that is, the distance between adjacent knots was exactly $T_{\max}/L$.

All the hyperparameters, for both DDH and DDH‐SMOL, were tuned using random search [53], a simple yet effective strategy for tuning hyperparameters. Unlike grid search, which exhaustively evaluates all possible combinations of hyperparameters, random search samples a much smaller number at random uniformly from the full hyperparameter space. This strategy has been shown to outperform grid search, particularly when only a subset of hyperparameters has a substantial impact on model performance. Its efficiency stems from the ability to explore a broader and more diverse region of the hyperparameter space within a constrained evaluation budget [54].

### 4.4 Evaluation

We applied DDH‐SMOL to the individual‐level risk prediction of ASCVD. The performance of our proposed method was then compared with benchmarks in terms of time‐dependent discriminative metric. The discriminative performance of survival models is primarily evaluated by the original concordance index (C‐index) [55] and its variants, assuming that subjects who are free of the event should be assigned a lower risk (or higher survival probability) than those who are not. Given the prediction time t (i.e., the time point when the dynamic prediction is made) and evaluation time s (i.e., the future time when the discriminative performance is of interest), the C‐index evolved into a time‐dependent version:

$$C(s|t)=\frac{\begin{array}{l} \sum_{1\leq i_{1},i_{2}\leq n}1\{\hat{S}(s|y_{i_{1}}(t),t)<\hat{S}(s|y_{i_{2}}(t),t),\delta_{i_{1}}=1,\\ t<{\tilde{T}}_{i_{1}}\leq s,{\tilde{T}}_{i_{1}}<{\tilde{T}}_{i_{2}}\} \end{array}}{\sum_{1\leq i_{1},i_{2}\leq n}1(\delta_{i_{1}}=1,t<{\tilde{T}}_{i_{1}}\leq s,{\tilde{T}}_{i_{1}}<{\tilde{T}}_{i_{2}})}.$$

Assessing how well a survival model could order risks up to time s, C(s|t) was hereafter utilized in comparing the discriminative performance of various methods. Means and standard deviations of C(s|t) were calculated across 20 random 80:20 splits. More specifically, for each split, subjects were randomly assigned to a training/validation set (80%) and a testing set (20%). Within each training/validation set, 20% of the subjects were randomly selected and reserved as the validation set for hyperparameter optimization.

### 4.5 Results

Means and standard deviations (all scaled by a factor of 100) of C(s|t) were reported in Table 2, where both the prediction time t and evaluation time s were in follow‐up years. Note that the three dynamic risk prediction models (JM, DDH, and DDH‐SMOL) tend to have superior performance than the non‐dynamic model based on the CPH, especially for t=10 when more longitudinal data were used for risk prediction. Among the three dynamic risk prediction models, DDH‐SMOL achieved the best performance in most cases. Notably, DDH‐SMOL significantly outperformed all the benchmarks when t=10 and s=20 (corresponding to a relatively long‐term prediction based on an extended longitudinal history); see the last column of Table 2. A direct comparison between the last column and “DDH” column of Table 2 clearly showed that DDH‐SMOL outperformed DDH in all cases, demonstrating the added value of the proposed SMOL. That is, replacing the original output layer with SMOL contributed positively to the neural network in predicting ASCVD risk.

**TABLE 2:** Average/standard deviation (×100) of C(s|t) evaluating the application to the cardiovascular risk prediction. The higher C(s|t), the better, for fixed s (the year when the prediction was issued) and t (the time length of utilized medical history). Notably, the last four columns were all associated with DDH‐SMOL: the rightmost column represented the full version of DDH‐SMOL, which retained all penalty terms in the loss (13); the remaining three corresponded to ablated versions, each omitting one penalty term.

t	s	Dataset	CPH	JM	DDH	DDH‐SMOL			
						($\lambda_{1}=0$)	($\lambda_{2}=0$)	($\lambda_{3}=0$)	
5	10	White female	78.8 (1.5)	80.4 (1.8)	80.5 (1.0)	79.8 (1.4)	80.0 (1.7)	80.3 (1.0)	80.6 (1.0)
		White male	75.4 (1.7)	76.4 (2.0)	73.3 (1.4)	72.4 (1.7)	72.1 (1.7)	72.3 (1.9)	73.4 (2.2)
		Black female	78.2 (3.5)	82.1 (3.5)	80.7 (3.0)	80.6 (2.3)	80.8 (1.9)	80.8 (2.2)	81.0 (2.6)
		Black male	72.7 (3.0)	74.1 (3.4)	72.7 (2.1)	72.1 (3.7)	71.8 (3.6)	72.3 (2.6)	74.2 (2.5)
5	15	White female	78.6 (1.3)	78.9 (1.9)	81.8 (1.3)	81.7 (1.4)	81.8 (1.7)	81.5 (1.5)	82.1 (1.3)
		White male	74.6 (1.1)	74.9 (1.1)	74.8 (2.1)	73.9 (1.5)	73.4 (1.6)	73.3 (2.1)	75.4 (2.4)
		Black female	77.0 (3.2)	80.1 (2.6)	81.4 (2.7)	82.2 (2.3)	82.4 (1.7)	82.3 (2.0)	82.4 (2.6)
		Black male	72.4 (3.3)	73.3 (2.6)	73.0 (2.9)	71.7 (2.9)	71.7 (2.7)	72.5 (3.9)	73.2 (1.5)
10	15	White female	74.6 (1.2)	79.3 (1.6)	79.3 (1.5)	79.3 (1.3)	79.5 (1.1)	79.7 (1.5)	80.1 (1.3)
		White male	69.3 (1.3)	75.3 (1.7)	74.2 (2.4)	75.8 (1.7)	74.6 (2.8)	73.9 (2.2)	76.0 (2.3)
		Black female	74.8 (2.4)	78.0 (3.6)	80.9 (2.8)	81.2 (2.2)	80.5 (3.2)	81.7 (2.7)	81.9 (2.6)
		Black male	65.1 (3.7)	75.9 (2.9)	73.8 (3.3)	74.3 (3.3)	73.4 (2.3)	73.6 (2.7)	77.6 (2.1)
10	20	White female	75.2 (1.1)	78.8 (1.4)	80.4 (1.7)	80.8 (1.6)	80.8 (1.7)	80.8 (1.5)	82.0 (1.4)
		White male	70.6 (1.3)	74.4 (1.6)	75.7 (3.0)	76.9 (1.6)	75.2 (2.7)	75.7 (2.4)	78.7 (2.1)
		Black female	74.9 (2.4)	76.7 (3.3)	82.3 (2.9)	82.7 (2.0)	82.3 (2.9)	83.0 (2.2)	83.5 (2.5)
		Black male	67.1 (3.3)	75.3 (3.0)	73.1 (4.1)	74.0 (3.1)	73.3 (2.2)	73.5 (3.3)	77.0 (1.9)

Compared with DDH, the usage of SMOL introduces several new hyper‐parameters. In 90% of our repeated runs, the selected value of L ranged from 10 to 100, with 10 and 30 being the most frequently chosen. The value of p was selected from the set {3,5,7,9} with approximately uniform frequency, although cubic B‐splines (p=3) were slightly favored by the resulting models. Accordingly, when using cubic B‐splines for this task, the total number of evenly spaced knots (L+2p+1) is recommended to fall within the range of 20 to 40.

We conducted additional analyses to demonstrate the necessity of each of the three penalty terms ${𝒫}_{1}$, ${𝒫}_{2}$ and ${𝒫}_{3}$ in loss function $\tilde{ℒ}$ (13). The columns of “$\lambda_{1}=0$”, “$\lambda_{2}=0$”, and “$\lambda_{3}=0$” under DDH‐SMOL in Table 2 summarized the performance in C‐index when ${𝒫}_{1}$, ${𝒫}_{2}$ and ${𝒫}_{3}$ were dropped, respectively. From the last four columns of Table 2, when any of these three penalty terms was dropped (i.e., setting $\lambda_{1}$, $\lambda_{2}$ or $\lambda_{3}$ to zero), we observed a uniform degeneration of the model performance in C‐index.

## 5 Simulation Study

### 5.1 Setting

We considered three simulated scenarios, each defined by a specific combination of longitudinal and survival submodels. Among these three, Scenario I followed submodels

(15) $$\begin{array}{ll} x_{i,j,k} & =\beta_{0,k}+b_{0,i,k}+(\beta_{1,k}+b_{1,i,k})\tau_{i,j}+\epsilon_{i,j,k},\\ & \;k=1,\ldots ,3,\;j=1,\ldots ,J_{i}, \end{array}$$

(16) $$\begin{array}{ll} h_{i}(t) & =h_{0}(t)\exp\left[\gamma^{⊤}z_{i}+\overset{3}{\underset{k=1}{\sum }}\alpha_{k}\left\{\beta_{0,k}+b_{0,i,k}+(\beta_{1,k}+b_{1,i,k})t\right\}\right], \end{array}$$

where regression coefficients $\beta_{0,k}$, $\beta_{1,k}$, $\alpha_{1}$, $\alpha_{2}$, $\alpha_{3}\in \mathbb{R}$ and $\gamma \in {\mathbb{R}}^{4}$ were all fixed; the baseline hazard $h_{0}(t)$ was a linear combination of B‐splines; Error terms $\epsilon_{i,j,k}$ ($∼N(0,\sigma_{e,k}^{2})$) were independent across all indices. In addition, subject‐specific random effects ${\left[b_{0,i,1},b_{1,i,1},b_{0,i,2},b_{1,i,2},b_{0,i,3},b_{1,i,3}\right]}^{⊤}\in {\mathbb{R}}^{6}$ were independent across subjects and were normally distributed with zero mean and covariance matrix $\sum_{b}\in {\mathbb{R}}^{6\times 6}$. Please refer to Table 3 for values of these components. Scenario II used the same longitudinal submodel (15) as Scenario I, but adopted a different survival submodel

(17) $$\begin{array}{ll} h_{i}(t) & =h_{0}(t)\exp\left\{\gamma^{⊤}z_{i}+\overset{3}{\underset{k=1}{\sum }}\alpha_{k}\left(\beta_{0,k}+\beta_{1,k}t\right.\right.\left.\left.+\sqrt{\left|b_{0,i,k}+b_{1,i,k}t\right|}\right)\right\}. \end{array}$$

Similarly, Scenario III retained (16) as the survival submodel but incorporated a nonlinear term of time into the longitudinal submodel, i.e.,

(18) $$\begin{array}{ll} x_{i,j,k} & =\beta_{0,k}+b_{0,i,k}+\beta_{1,k}\tau_{i,j}+b_{1,i,k}\tau_{i,j}^{2}/10+\epsilon_{i,j,k},\\ & \;k=1,\ldots ,3,\;j=1,\ldots ,J_{i}. \end{array}$$

**TABLE 3:** Values of components in generating simulative data.

Parameters	Values
n	5000
$\beta_{0,1},\beta_{0,2},\beta_{0,3}$	0.2
$\beta_{1,1},\beta_{1,2},\beta_{1,3}$	0.1
$\alpha_{1},\alpha_{2},\alpha_{3}$	0.2
$\sigma_{e,1},\sigma_{e,2},\sigma_{e,3}$	$\sqrt{0.8}$
γ	${\left[0.2,0.2,0.2,0.2\right]}^{⊤}$
$h_{0}(t)$	$\sum_{\ell }(-3)\times B_{\ell ,3,\xi }(t)$ with ξ={−1,−1,0,3,6,9,12,15,18,21,24,27,30,31,31}
$\sum_{b}$	$0.8\times I_{6}+0.1\times 1_{6\times 6}$ with $I_{6}$ as the 6×6 identity matrix and $1_{6\times 6}$ as the 6×6 matrix of ones

For each scenario, we generated 20 synthetic datasets by varying the random seed. Taking use of values in Table 3, the data generation proceeded as follows:

1. We assumed that all participants were scheduled to revisit every two years. However, each scheduled visit had a 50% probability of being missed. Given the finalized visit times, time‐varying covariates were simulated according to submodel (15) (for Scenarios I and II) or (19) (for Scenario III).
2. Times to events, $T_{i}$, were drawn using the inverse transform sampling, based on the survival submodel (16) (for Scenarios I and III) or (18) (for Scenario II). Censoring times were drawn independently from an exponential distribution.

### 5.2 Results

We applied DDH‐SMOL along with benchmarks (as described in Section 4.2) to each simulated dataset. For each simulated dataset, subjects were randomly assigned to a training/validation set (80%) and a testing set (20%). Within each training/validation set, 20% of the subjects were randomly selected and reserved as the validation set for hyperparameter optimization. The results were summarized in Table 4. Note that for all three simulation scenarios, we consistently fit the joint model of (15) and (16) for the JM approach.

**TABLE 4:** Average/standard deviation (×100) of C(s|t) evaluating the performance of distinct approaches in the simulation study. The higher C(s|t), the better, fixing s (the year when the prediction was issued) and t (the time length of utilized medical history).

t	s	Scenario	CPH	JM	DDH	DDH‐ SMOL
2	4	I	69.3 (1.7)	78.5 (1.8)	75.6 (2.3)	77.1 (2.6)
		II	56.0 (1.1)	57.3 (2.2)	74.6 (2.4)	75.2 (1.7)
		III	64.5 (1.6)	51.6 (1.9)	72.7 (2.5)	74.5 (2.5)
2	6	I	70.0 (1.7)	80.0 (1.4)	76.9 (2.3)	77.3 (2.4)
		II	56.3 (1.0)	56.8 (1.6)	74.8 (2.1)	75.0 (1.9)
		III	64.1 (1.5)	62.2 (1.8)	75.3 (1.9)	76.3 (1.8)
4	6	I	72.5 (1.5)	83.5 (1.5)	80.3 (2.1)	81.0 (2.0)
		II	51.7 (1.5)	55.8 (2.1)	78.8 (2.1)	80.4 (2.0)
		III	65.2 (1.7)	70.6 (1.9)	81.5 (2.0)	82.4 (1.9)
4	8	I	73.2 (1.4)	84.2 (1.2)	80.6 (1.3)	82.1 (1.4)
		II	53.2 (1.6)	55.5 (1.7)	78.7 (1.6)	80.5 (2.1)
		III	65.6 (1.8)	73.0 (1.6)	80.2 (1.9)	81.7 (2.2)

Consistent with our findings in Section 4.5, the outputs from DDH methods were more stable across all three scenarios. Their performance was largely unaffected by nonlinear terms in submodels. Moreover, DDH‐SMOL consistently enhanced DDH's predictive accuracy, particularly when predictions were issued at later time points (i.e., t=4), highlighting its advantage in leveraging more follow‐up measurements.

The DDH‐SMOL achieved performance comparable to that of the JM approach in Scenario I, where the true model was used for the JM. However, in Scenarios II and III, where nonlinear terms were introduced into the survival or longitudinal submodels, the DDH‐SMOL significantly outperformed the JM. In particular, in Scenario III, JM performed even worse than the non‐dynamic CPH at t=2. This was likely due to a combination of model misspecification and limited historical information available at early prediction times, which together constrained JM's predictive accuracy.

## 6 Discussion

This paper introduces SMOL, an innovative output layer for neural networks that enforces both differentiability and monotonicity in estimating functions. To illustrate its practical utility, we concatenate SMOL to DDH, an existing competitive deep learning model for dynamic risk prediction. The resulting model, DDH‐SMOL, was applied to LRPP data to approximate personalized survival functions based on historical longitudinal observations. Our numerical study demonstrates that integrating SMOL tends to improve predictive accuracy, particularly when more longitudinal data are available.

A key advantage of SMOL lies in its compatibility with existing deep learning models: architecturally, SMOL can simply replace the original output layer, requiring no modification to the internal network structure. This flexibility offers two major benefits. First, as deep learning models continue to be developed in dynamic risk prediction, SMOL can be seamlessly incorporated into a wide variety of models, enabling further gains in predictive performance. Second, SMOL is flexible with input modality of neural networks. Although our study centers on sparsely observed longitudinal data, SMOL remains applicable for estimating survival functions in any setting where the input information can be extracted, processed and eventually represented as a vector. These two benefits make SMOL highly adaptable and scalable. In practice, SMOL can be integrated into models that process multi‐modal biomedical data, such as genomic sequences, medical imaging, and electronic health records. The resulting models can deliver individualized survival predictions that dynamically update as various new data become available. Clinicians can leverage these continuously updated risk estimates to make more informed and timely decisions, adjust treatment strategies, and ultimately improve patient outcomes. Despite our current focus on survival prediction with a single terminal event, SMOL can generalize to more complex event structures (such as competing risks and multi‐state processes), thus providing clinicians with tools to look into patients' risks for multiple diseases simultaneously.

## Funding

This work was supported by the National Institutes of Health (Grant No. R01HL136942, R21EY035296, R01CA289249).

## Conflicts of Interest

The authors declare no conflicts of interest.

## Data Availability

LRPP data used in this study are not publicly available due to the data usage agreement with the National Heart, Lung, and Blood Institute (NHLBI). These data can be requested from the Biologic Specimen and Data Repositories Information Coordinating Center of NHLBI.

## Appendix A B‐Splines

Splines are functions constructed by smoothly joining together polynomial segments. They are widely used across various branches of science and engineering [56]. In survival analysis, splines have become a well‐established tool for representing unknown smooth functions, such as baseline hazard function [57] and time‐dependent effects [58].

Among the many types of splines, B‐splines are especially popular due to a key theoretical property: any spline can be expressed as a linear combination of B‐splines [59]. This makes B‐splines highly suitable for function approximation. Specifically, B‐splines are defined by a degree $p\in {\mathbb{Z}}^{+}$ and an ascending knot sequence ξ, which specifies the locations where polynomial segments join. Given p and $\xi =\{\xi_{1},\ldots ,\xi_{L+2p+1}\}$, the ℓth B‐spline, say $B_{\ell ,p,\xi }(u)$, is zero if $\xi_{\ell }=\xi_{\ell +p+1}$ and otherwise defined recursively via the Cox‐de Boor formula [43, 60]:

$$B_{\ell ,p,\xi }(u)=\frac{u-\xi_{\ell }}{\xi_{\ell +p}-\xi_{\ell }}B_{\ell ,p-1,\xi }(u)+\frac{\xi_{\ell +p+1}-u}{\xi_{\ell +p+1}-\xi_{\ell +1}}B_{\ell +1,p-1,\xi }(u),$$

where $\xi_{\ell }$ is the ℓth entry of ξ. This recursion starts with

$$B_{\ell ,0,\xi }(u)=1(u\in [\xi_{\ell },\xi_{\ell +1})).$$

A notable feature of B‐splines is the local support: each B‐spline is positive only within a finite interval and zero elsewhere. This property contributes to their near‐orthogonality, improves numerical stability, and facilitates efficient computation.

## Appendix B Proofs

Proof of Proposition 1 Recall $O_{\text{SMOL}}(u;o_{i,t}^{\text{FNN}},B_{L,p,\xi })$ in (10):

$$O_{\text{SMOL}}(u;o_{i,t}^{\text{FNN}},B_{L,p,\xi })=\overset{L+p}{\underset{\ell =1}{\sum }}\left\{\overset{\ell }{\underset{k=1}{\sum }}\phi (o_{i,t,k}^{\text{FNN}})\right\}B_{\ell ,p,\xi }(u).$$

For $u\in [\xi_{p+1},\xi_{L+p+1}]=[0,T_{\max}]$,

$$\frac{\partial }{\partial u}O_{\text{SMOL}}(u;o_{i,t}^{\text{FNN}},B_{L,p,\xi })=p\overset{L+p}{\underset{\ell =2}{\sum }}\frac{\phi (o_{i,t,\ell }^{\text{FNN}})}{\xi_{\ell +p}-\xi_{\ell }}B_{\ell ,p-1,\xi }(u).\;[45]$$

We complete the proof by noting that $\phi (o_{i,t,\ell }^{\text{FNN}})>0$ for ℓ≥2 and $B_{\ell ,p-1,\xi }(u)$ is positive for $u\in (\xi_{\ell },\xi_{\ell +p})$ and nonnegative elsewhere [45].

## Appendix C Computing Environment

All experiments were conducted on a server equipped with dual Intel Xeon Gold 5218 and three NVIDIA Quadro RTX 8000 graphics processing units. The code for DDH and DDH‐SMOL was executed within a Conda [61] environment with Python 2.7.15 [62] and TensorFlow 1.15.0 [63].

## Appendix D Data Availability

LRPP data used in this study are not publicly available due to the data usage agreement with the National Heart, Lung, and Blood Institute (NHLBI). These data can be requested from the Biologic Specimen and Data Repositories Information Coordinating Center of NHLBI.

## Appendix E Code Accessibility

The Python code of DDH‐SMOL is accessible at https://github.com/ZhiyangGeeZhou/DDH‐SMOL.
